# Neurochemical and genetic factors in panic disorder: a systematic review

**DOI:** 10.1038/s41398-024-02966-0

**Published:** 2024-07-18

**Authors:** Adriana Carvalho Natal Moraes, Clarissa Wijaya, Rafael Freire, Laiana Azevedo Quagliato, Antonio Egidio Nardi, Peter Kyriakoulis

**Affiliations:** 1 Federal University of Rio de Janeiro, Rio de Janeiro, Brazil; 2 School of Psychology, Swinburne University, Melbourne, VIC Australia; 3 Department of Psychiatry and Centre for Neuroscience Studies, Queen’s University, Kingston, ON Canada

**Keywords:** Human behaviour, Epigenetics and behaviour

## Abstract

This systematic review addresses the complex nature of Panic Disorder (PD), characterized by recurrent episodes of acute fear, with a focus on updating and consolidating knowledge regarding neurochemical, genetic, and epigenetic factors associated with PD. Utilizing the PRISMA methodology, 33 original peer-reviewed studies were identified, comprising 6 studies related to human neurochemicals, 10 related to human genetic or epigenetic alterations, and 17 animal studies. The review reveals patterns of altered expression in various biological systems, including neurotransmission, the Hypothalamic-Pituitary-Adrenal (HPA) axis, neuroplasticity, and genetic and epigenetic factors leading to neuroanatomical modifications. Noteworthy findings include lower receptor binding of GABAA and serotonin neurotransmitters in the amygdala. The involvement of orexin (ORX) neurons in the dorsomedial/perifornical region in triggering panic reactions is highlighted, with systemic ORX-1 receptor antagonists blocking panic responses. Elevated Interleukin 6 and leptin levels in PD patients suggest potential connections between stress-induced inflammatory changes and PD. Brain-derived neurotrophic factor (BDNF) and tyrosine receptor kinase B (TrkB) signaling are implicated in panic-like responses, particularly in the dorsal periaqueductal gray (dPAG), where BDNF’s panicolytic-like effects operate through GABAA-dependent mechanisms. GABAergic neurons’ inhibitory influence on dorsomedial and posterior hypothalamus nuclei is identified, potentially reducing the excitability of neurons involved in panic-like responses. The dorsomedial hypothalamus (DMH) is highlighted as a specific hypothalamic nucleus relevant to the genesis and maintenance of panic disorder. Altered brain lactate and glutamate concentrations, along with identified genetic polymorphisms linked to PD, further contribute to the intricate neurochemical landscape associated with the disorder. The review underscores the potential impact of neurochemical, genetic, and epigenetic factors on the development and expression of PD. The comprehensive insights provided by this systematic review contribute to advancing our understanding of the multifaceted nature of Panic Disorder and pave the way for targeted therapeutic strategies.

## Introduction

Panic disorder (PD) is an intricate and incapacitating psychiatric condition characterized by recurrent episodes of acute fear, with a lifetime prevalence of 4.7% [1], associated with substantial individual, social, and economic costs [2, 3]. Currently, it is acknowledged that when an individual perceives a stimulus as potentially threatening, a combination of adaptive changes involving neurochemical, neuroendocrine, and behavioral responses are activated to enhance the chances of survival. These neurobiological fear responses constitute the fear circuitry, encompassing the amygdala, thalamus, hippocampus, insula, and prefrontal cortex [4]. Nevertheless, the pathophysiology of PD remains poorly understood despite advancements in treatment and diagnosis [5].

Genetic and epigenetic factors, have played a pivotal role in comprehending the mechanisms and etiology of PD, contributing to the present understanding of the fear circuitry in the brain [6]. While the precise neural pathways triggering panic attacks remain indeterminate [7], a combination of complex fear and anxiety circuitry is hypothesized to be involved [8].

This systematic review aims to update and consolidate the knowledge regarding PD, focusing on neurochemical, genetic, and epigenetic factors. The primary objectives are: (1) understanding the interplay of neurocircuitry and neurochemical factors in the pathophysiology of PD, (2) identifying recent advances in genetic research related to the development and maintenance of PD, and (3) establishing foundations and new insights into the fear circuitry involved in PD. The secondary objective is to identify sophisticated translational models to assess the empirical value of animal research in understanding the neurobiological basis and pathophysiology of PD.

A prominent biological theory shedding light on the etiology of PD proposes that panic symptoms result from an imbalance in one or more neurotransmitters, including serotonin, norepinephrine, dopamine, and gamma-aminobutyric acid (GABA) [9]. Support for this theory is evidenced by the reduction of symptoms experienced by many PD patients when using antidepressant or anxiolytic medication [10]. Neurochemical theories suggest dysregulated functioning of neurotransmitters, such as a deficiency in the serotonergic system or excess serotonin [11]. Additionally, abnormal chemoreceptor reactivity may be implicated in the etiology of PD, with inhibitory and excitatory neurotransmitters like glutamate, gamma-amino butyric acid, cholecystokinin, adenosine, dopamine, and norepinephrine potentially playing a role in panic regulation [12].

Other neurochemical theories pinpoint a dense concentration of neuropeptide Y (NPY) in anxiety circuits, believed to be involved in fear memory consolidation [13]. Reduced concentrations of NPY in patients with PD make them more susceptible to stress and fear responses [14].

Genetic studies form a significant aspect of biological theories, proposing an inherited genetic predisposition in individuals with PD. Crowe and colleagues observed a prevalence of PD among families of PD patients, with ~25% of first-degree relatives receiving a PD diagnosis. Evidence also suggests up to a threefold increase in prevalence among first-degree relatives of PD patients [15–17]. The respiratory subtype of PD has demonstrated a higher familial prevalence in comparison to the non-respiratory subtype in several studies [18–20]. Although twin studies have provided valuable insights, suggesting a link between genetic factors and the pathogenesis of PD with moderate heritability contributions [21, 22], criticisms include the failure to identify a mode of inheritance aligning with Mendelian patterns. This points to a complex genetic inheritance model involving interactions among multiple vulnerabilities and genes [23]. To date, only a limited number of risk genes have been identified, and little is known about gene-environment interactions specific to PD [24].

Here we choose the period covered between 2010 and 2020 (10 years) encompasses a bibliography that is already consolidated and debated within the community of the field. These are works of significance, cited, and already assessed by the community. The exclusion of more recent years (the last 3 years) aims to avoid bibliographies that are not yet fully matured, with lower citations and still-preliminary analyses by their scientific peers. We understand that this temporal safety margin allows for a review with greater support and relevance.

## Methods

The systematic review was conducted according to the PRISMA guidelines. This review draws on literature found in articles via PubMed Database. Limits were applied to English language, 2010–2020, and clinical studies and a randomized controlled trial. The keyword search includes panic disorder*(serotonin/noradrenalin/biomarker/corticotropin releasing* OR CRF OR CRH/functional near infrared spectroscopy OR fNIRS/angiotensin II type 1 receptor OR AT1R). In the first step of the process, titles and abstract were manually screened against the inclusion/exclusion criteria. At this stage, retained articles were assessed against the following inclusion criteria: (1) an original research paper, (2) focused specifically on PD with/without comorbidity, (3) focused on the panic/fear circuitry, and (4) adult participants studies. Articles are excluded if they: (1) are meta-analysis/systematic review/theoretical literature, (2) unrelated to PD, (3) focused on the therapy modalities/pharmacological intervention of PD, and (4) articles have no abstracts.

**Fig. 1:**
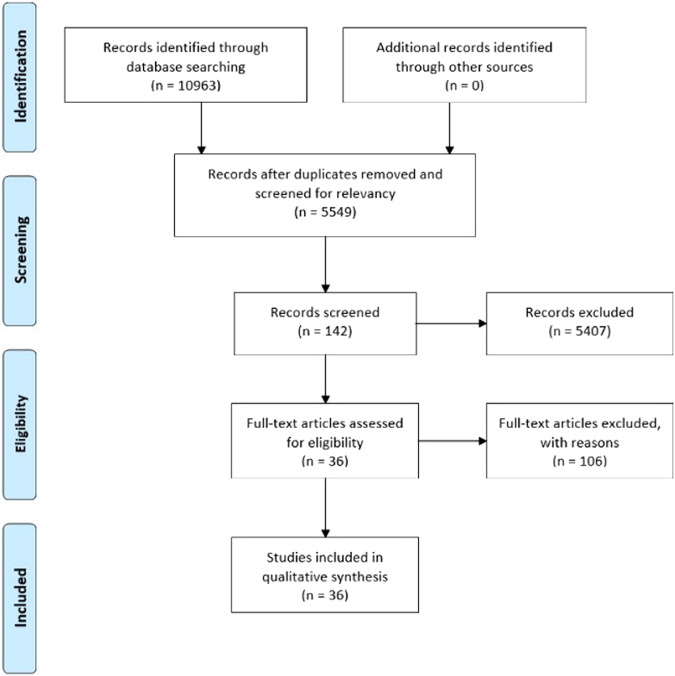
Selection criteria. PRISMA flow chart of study identification and selection process.

At the next step, full-text articles were screened for their eligibility for qualitative synthesis. The article inclusion process was conducted independently by two reviewers, PK and CW, with a third review, RF, involved to resolve any inclusion disagreement before proceeding. Articles that are satisfactory will be included in the synthesis (Fig. 1).

## Results

The keyword search generated a total of 10,963 articles. Following the removal of duplicates and irrelevant articles, 5549 articles were retained for abstract screening. The exclusion criteria removed 5407 articles and 143 articles were retained for the full-text assessment. In the full-text screening stage, 106 articles were excluded, and 36 articles were included in the qualitative data synthesis. The 36 articles were analyzed, and findings were synthesized into neurochemical studies, genetics, and epigenetics studies with primary focus of explaining PD etiology.

### Neurochemical studies

The database search identified six human neurochemical studies that met the inclusion criteria. The results of these human studies are presented in Table 1. The seven studies investigated various neurochemical types and their impact or lack thereof on panic disorder (PD) based on the respective study interventions.

**Table 1:** Summary of neurochemical studies.

Study	Sample	Measurement	Intervention	Findings	*P* value	Comment
Maddock et al. [25]	N = 34 participants n = 22 patients (13 remitted, 8 symptomatic) n = 12 HC	MRI, 1H-MRS	Vigilance task	Sig. increase in visual cortex lactate/NAA during visual stimulation compared with baseline across groups	<0.0001	Matched for gender, age, and education. Remitted PD patients were free of panic attacks and other significant PD symptoms for at least 4 months. Subjects were evaluated by a psychiatrist with the Structured Clinical Interview for DSM-IV.
				PD > HC, sig. main effect of group for percentage change from baseline lactate/NAA during visual stimulation and subsequent recovery periods.	0.0018	
				Sig. main effect of time for mean lactate double peaks before and during visual stimulation.		
Zwanzger et al. [26]	N = 18 HC right-handed male subjects (26.9 years ± 4.5 years)	H-MRS	CCK-4 challenge	Sig. increase in panic symptoms as indicated by increased between the API scores	<0.0001	All subjects had to be free of any medication. Any drug intake was ruled out by urine toxicology screening.”
				Sig. increase in panic symptoms as indicated by increased between the PSS scores	<0.0001	
				Sig. increase in HR in experimentally induced panic	<0.0001	
				Glutamatergic baseline concentration seems to mainly determine the extent of percentage of Glx/Cr increase	<0.0001	
				Sig. increase in CCK-4-related HPA axis stimulation mirrored by a significant effect on plasma cortisol levels 10–15 min after CCK-4 administration	0.0002	
				Sig. positive correlation between maximum Glx/Cr concentrations and API max	0.0090	No effects were found for the correlation between maximum Glx/Cr concentrations and API max
				Sig. positive correlation between baseline Glx/Cr concentrations and HR max	0.0270	
Mueller et al. [29]	N = 66 n = 22 PD n = 21 MD n = 23 HC	EEG	Gambling task	N300H latencies did not differ between groups	>0.05	
				PD > HC, N300H magnitudes significantly higher	<0.02	
				PD without MD comorbidity > HC, N300H magnitudes remained significantly higher	0.0100	
				PD > MD, temporal N300H spread significantly different	<0.05	
				PD > HC, temporal N300H spread significantly different	<0.005	
				PD/MD treated with SRIs > PD/MD not treated with SRI at the time of the study, showed significantly larger N300H spread	<0.014	
				PD > HC, P300 significantly increased	<0.01	
				Group x peak cardiac acceleration, no main effect or interaction	>0.2	
				PD > HC, significantly greater N300H when controlling for P300 amplitude	<0.03	
Jakuszkowiak-Wojten et al. [30]	N = 28 n = 14 psychotropic drug=native outpatients with PD without agoraphobia n = 14 HC	Saliva samples	None	No. sig difference in salivary cortisol between PD and HC at awakening	0.7700	
				No. sig difference in salivary cortisol between PD and HC at awakening + 15 min	0.4260	
				No. sig difference in salivary cortisol between PD and HC at awakening + 30 min	0.5140	
				No sig. difference between PD and HC with changes in time and CAR parameters		
				No sig. difference in AUCg (nmol/L*min) between PD and HC	0.5610	
				No sig. difference in AUCi (nmol/L*min) between PD and HC	0.1380	
Wichmann et al. [31]	n = 30 PD/AG n = 4 PD without AG n = 34 HC	CBT	DEX-CRH test CBT for patient group	Between groups, no sig. difference in DEX-CRH suppression rate	1.0000	
				At cortisol levels baseline, PD < HC, sig. difference of cortisol levels measured 75 min prior to CRH-injection	0.0160	
				At cortisol levels baseline, PD < HC, sig. difference of cortisol levels measured 1 min prior to CRH-injection	0.0400	
				At ACTH baseline, no sig group differences in 75 min prior to the CRH-injection		
				At ACTH baseline, no sig group differences in 1 min prior to the CRH-injection		
				At baseline, sig. main effect of time for cortisol levels and the BDI sum score.	≤0.001	
				No sig. time x group interaction effect	0.4320	
				No sig. main effect of group	0.7670	
				At ACTH concentration baseline and the BDI sum score, sig. main effect of time is observed	≤0.001	
				At ACTH concentration baseline and the BDI sum score, no time x group interaction effect	0.5970	
				At ACTH concentration baseline and the BDI sum score, no main effect of group	0.8090	
				With psychotherapy, sig. correlation between mean cortisol concentrations and ACQ loss of control outcome score	0.0050	
				With psychotherapy, no sig. correlation between mean cortisol concentrations and PAS, ACQ physical concerns and total score, BSQ and MI outcome scores	≥0.028	
Kim et al. [32]			IL6	MDD, PD > HC at baseline levels were higher	0.022	
				MDD, PD > HC at 3rd visit has levels were higher	0.003	
				PD > MDD, HC at the 4th visit, IL-6 levels were significantly higher	0.016	
				In PD, significantly negative association with treatment response	0.022	
			Leptin	MDD, PD > HC showed significantly higher levels in 3rd visit	0.032	
				MDD, PD > HC showed significantly higher levels in 4th visit	0.003	

*API* Acute Panic Inventory, *PSS* Panic Symptom Scale, *Glx/Cre* glutamate + glutamine/creatine, *EEG* electroencephalogram, *N300H* centromedial brain activity 300 ms after presentation of motivationally meaningful stimuli, *CAR* cortisol awakening response, *AUCg* area under curve with respect to the ground, *AUCi* area under curve with respect to the increase, *CCK4* cholecystokinin-tetrapeptide, *SRI* serotonin reuptake inhibitors, *BDI* Beck’s Depression Inventory, *ACQ* Agoraphobic Cognitions Questionnaire, *PAS* Panic and Agoraphobia Scale, *BSQ* Body Sensation Questionnaire, *MI* Mobility Inventory, *ACTH* adrenocorticotropic hormone.

A study regarding the activity-dependent changes in brain lactate and glutamate + glutamine (glx) in the visual cortex was performed involving 21 PD patients (13 remitted, 8 symptomatic) and 12 healthy controls (HC) [25]. PD patients exhibited higher activity-dependent increases in brain lactate compared to their healthy counterparts (p = 0.0018). Conversely, activity-dependent alterations in glutamate and glutamine (glx) were reduced in PD patients in comparison to the control group. Moreover, the temporal correlation between lactate and glx changes demonstrated a significantly stronger association in control subjects than in PD patients. This groundbreaking evidence contradicts the prevailing model proposing a general enhancement of activity-dependent metabolic responses in the PD context.

The fact that glx responses are diminished and temporally uncoupled from lactate responses in PD challenges the notion of a pervasive upregulation of activity-dependent brain metabolic responses in this condition. The heightened accumulation of activity-dependent brain lactate emerges as a distinctive trait feature of PD. Considering the intimate interplay between lactate and pH in the brain, these findings align with a model positing dysregulation in brain metabolism and pH, potentially linked to the altered functionality of acid-sensitive fear circuits. Such dysregulation contributes to the enduring susceptibility observed in PD.

Increasing evidence suggests that gamma-amino-butyric-acid (GABA) mediated neurotransmission plays a role in the pathophysiology of panic disorder (PD). In addition, derivates of progesterone and deoxycorticosterone modulate neuronal excitability through GABAA receptor interactions. The evaluation of glutamate concentration was conducted through proton magnetic resonance spectroscopy (H-MRS) after panic induction via cholecystokinin-tetrapeptide (CCK-4) challenge and treatment with tiagabine within a cohort of healthy participants [26]. A notable finding emerged, underscoring the pivotal role of the baseline glutamate concentration in determining the extent of Glx/Cr increase (p < 0.0001). Furthermore, the elevation in CCK-4-induced activation of the hypothalamic-pituitary-adrenal (HPA) axis relates to a significant increase of plasma cortisol levels ~10–15 min post CCK-4 administration (p = 0.0002) .Additionally, the minimum Glx/Cr concentration positive correlates with the maximum Agoraphobic Panic Inventory (API) scores (p = 0.009), as well as between baseline Glx/Cr concentrations and maximum heart rate (HR) (p = 0.027). Moreover, 3a, 5a-tetrahydro deoxycorticosterone (3a,5a-THDOC) increased following panic induction with CCK-4 (p = 0.005), and this elevation correlates with a reduction in panic symptoms during the CCK-4 challenge. 3a, 5a-tetrahydro deoxycorticosterone (3a,5a-THDOC), a neuroactive steroid, is a positive modulator of the GABAA receptor [27] and displays anxiolytic activity in several animal models. However, how neuroactive steroid compounds act in PD patients remains unknown.

Interactions of higher cortical processes and visceral, especially cardiac activity have been assumed to have an important role in panic attacks and PD [28]. As follows, the relationship between PD and serotonin reuptake inhibitors (SRIs) on the coupling of cortical and cardiac activity was evaluated [29]. Some intriguing findings suggested that PD patients exhibited significantly higher N300H magnitudes compared to HC (p < 0.02). This difference persisted when PD patients without major depression (MD) comorbidity were analyzed separately. Further analysis highlighted an overall difference in the spread of N300H in PD compared to both MD patients and HC. Consequently, brain–heart covariation is significantly elevated in individuals suffering from PD. Moreover, SRI treatment resulted in greater N300H activity spread in PD patients than in non-SRI treated PD patients. Therefore, PD and serotonin are involved in brain–heart interactions in PD patients.

The involvement of the hypothalamic-pituitary-adrenal axis in the pathophysiology of PD has been hypothesized. A study evaluating the cortisol awakening response (CAR) in drug-naïve PD patients [30] found no significant differences were found for CAR between PD and HC. In addition, no correlations were observed between CAR and anxiety measures in PD and HC.

Studies investigating the HPA axis responsivity with the DEX–CRH test in PD patients reported mixed findings. The DEX–CRH test requires the oral intake of a synthetic cortisol-like compound (dexamethasone). In healthy individuals, the prolonged administration of dexamethasone inhibits the adrenocorticotropic hormone (ACTH) and cortisol release. Therefore, the hormonal response to the DEX–CRH test provides an index for the normative functioning of the central glucocorticoid feedback regulation of the hypothalamic-pituitary-adrenal (HPA) axis. The stress hormone response to the DEX-CRH test in PD with or without agoraphobia was tested [31]. PD patients demonstrated greater cortisol baseline levels at two-time points (75 min and 1 min) before CRH injection compared to HC. However, there was no difference in adrenocorticotropin hormone (ACTH) levels at baseline between PD and HC before CRH injection at the two-time points. A significant main effect of time for cortisol levels and BDI sum score was noted. However, the main effect of the group was insignificant, and the time x group interaction effect was not detected. The only significant main effect observed was the time of ACTH concentration baseline and the BDI sum. With psychotherapy, a significant correlation was noted between mean cortisol concentrations and the ACQ loss of control outcome score, but not with other measures.

Even though accumulating evidence implicates inflammatory processes in the pathophysiology of PA and PD, few studies have investigated cytokine levels in individuals with PD. Accordingly, peripheral biomarkers in individuals with panic attacks (PA) in PD patients and major depressive disorder (MDD) patients were investigated [32]. The Panic Disorder Severity Scale (PDSS) was used as one of the clinical measurements for objective symptom severity. The results indicated interleukin 6 (IL-6) and leptin levels in PD and MDD patients increased at baseline, visit 3, and visit 4 of the PA treatment. PD patients demonstrated higher IL-6 levels at visit 4 than MDD and HC groups. Interestingly, IL-6 showed clinical correlation with PDSS items, despite its lack of predictive value.

### Genetics and epigenetics

The database search identified 10 human genetic/epigenetic studies that met the inclusion criteria, and the summary of these studies is presented in Table 2. The studies encompass investigations into genotypes, phenotypes, allelic variations, and gene methylation associated with panic disorder (PD). Notably, the focus of these genetic studies involves the neuropeptide S receptor gene (NPSR1), 5-Hydroxytryptamine Receptor 1 A (HTR1A) gene, and the norepinephrine transporter gene SLC6A2.

**Table 2:** The summary of genetic studies results.

Study	Gene	Polymorphism/ Methylation	Findings	*P*-value
Dannlowski et al. [35]	NPSR1	rs324981	TT, AT > AA, significant increased amygdala responsiveness to fear-relevant faces	<0.01
			T-alleles indicates strong association of amygdala responsiveness and harm avoidance	0.005
Gechter et al. [36]	NPSR1	rs324981	In perception phase, A/T > A/A showed higher amygdala activation during the perception of agoraphobia specific stimuli compared to neutral images	0.006
		rs324981	In perception phase, single or two T-alleles displayed the highest activation in the iOFC	0.054
Choi et al. [40]	HTR1A	rs6295	GG > CC genotype carriers has significantly higher PDSS scores for PD without agoraphobia	0.036
			G > C allele had significantly higher PDSS score in PD without agoraphobia	0.025
Yu et al. [41]	HTR1A	rs6295	GG/CG genotype, sig. positive correlation with APPQ interoceptive fear subscale scores	0.008
			GG/CG genotype, FA values of the cingulate gyrus process of the left cingulum showed sig. positive correlation with the APPQ interoceptive fear subscale and ASI-R fear of publicly observable anxiety reaction	0.001
			CC genotype, sig. positive correlation with APPQ	0.017
			CC genotype, sig. positive correlation with APPQ agoraphobia	0.029
			CC genotype, sig. positive correlation with APPQ social phobia	0.008
Straube et al. [42]	HTR1A	rs6295	GG > CC genotype carriers in PD/AG showed significantly associated with acute flight behavior before therapy	<0.05
			GG/CC and escapers and non-escapers, significant interaction effects between the two groups during anticipation and exposure period	<0.05
			CC escapers > non-escapers, significantly higher anticipatory anxiety during the anticipation period	<0.01
			CC escapers > G allele homozygous escapers, significantly higher anxiety	<0.05
Hommers et al. [46]	MIR579	rs2910931	Minor SLC6A3 T-allele upstream of MIR579 was associated with PD	0.004
			Minor SLC6A3 T-allele upstream of MIR579 was associated with higher anxiety trait in HC	0.047
			PD with two T-alleles showed elevated heart rates in anxiety-provoking BAT	0.005
			Homozygotic T-alleles carriers showed significantly higher heart rate increase from the last minute of anticipation to the first minute of the exposure phase irrespective of active avoidance behavior	0.005
Buttenschon et al. [45]	SLC6A2	rs2242446	Significantly associated with PD	<0.002
		rs11076111	Significantly associated with PD	0.0220
		rs747107	Significantly associated with PD	0.0016
		rs1532701	Significantly associated with PD	0.0385
		rs933555	Significantly associated with PD	0.0128
		rs16955584	Significantly associated with PD	0.0499
		rs36021	Significantly associated with PD	0.0434
		rs2242446	Significantly associated haplotype, primarily caused by differences in the frequencies of two haplotypes (T-T and C-T)	0.0022
		rs8052022	Significantly associated haplotype, primarily caused by differences in the frequencies of two haplotypes (T-T and C-T)	0.0022
			T-T haplotype appears to be a protective haplotype with frequencies of 0.689 and 0.780 among PD cases and controls, respectively	0.00005
			C-T haplotype appears to be a risk haplotype with frequencies 0.290 and 0.207 among PD cases and control, respectively.	0.0010
Ridderbusch et al. [49]	NOS1	NOS1 ex1f-VNTR	In PD/AG, S/S genotype showed significantly less activation of the right amygdala in early extinction	<0.001
			In PD/AG, S/S genotype showed significantly less activation of the right hippocampus in early extinction	0.004
			In PD/AG, S/S genotype showed significantly less activation of the left amygdala in early extinction both before and after CBT treatment	0.01
			Significant (PD/AG > HC) > (S/S > L/L) > (CS+ < CS-) interaction effect with increased left hippocampus activation in early extinction	0.007
			Significant (PD/AG > HC) > (S/S > L/L) > (CS+ > CS-) interaction effect with increased left amygdala activation in early extinction	0.13
			Significant (PD/AG > HC) > (S/S > L/L) > (CS+ > CS-) interaction effect with increased right amygdala activation in early extinction	0.11
			In PD/AG, (S/S > L/L) > (CS+ > CS-) interaction showed increased right amygdala activation in early extinction	0.011
			In PD/AG, (S/S > L/L) > (CS+ > CS-) interaction showed increased right amygdala activation in late extinction	0.004
			In PD/AG, (S/S > L/L) > (CS+ > CS-) interaction showed increased left hippocampus activation in late extinction	0.01
Bayoglu et al. [54]	ACE, ATR1	I/D, A1166C	No significant differences in genotype frequencies of functional ACE I/D and ATR1 A1166C gene polymorphisms between PD and HC	>0.05
	ACE	I/D	I-allele in the I/D polymorphism is more frequent compared to the controls in male subgroups of PD	0.032
Ziegler et al. [58]	MAOA	CpG3	PD < HC, sig. MAOA hypomethylation at baseline in discovery sample	<0.001
			In PD sample, responders > non-responders, increased average methylation after therapy for responders, dependent on responder status (according to the number of panic attacks)	0.001
		CpG4	In PD sample, responders > non-responders, increased average methylation after therapy for responders, dependent on responder status (according to the number of panic attacks)	0.003
		CpG6	PD < HC, sig. MAOA hypomethylation at baseline in discovery sample	<0.001
			In PD sample, responders > non-responders, increased average methylation after therapy for responders, dependent on responder status (according to the number of panic attacks)	0.003
		CpG7	PD < HC, sig. MAOA hypomethylation at baseline in discovery sample	<0.001
		CpG8	PD < HC, sig. MAOA hypomethylation at baseline in discovery sample	0.004
		CpG9	PD < HC, sig. MAOA hypomethylation at baseline in discovery sample	0.001
		CpG11	In PD sample, responders > non-responders, increased average methylation after therapy for responders, dependent on responder status (according to the number of panic attacks)	0.002
		CpG12	PD < HC, sig. MAOA hypomethylation at baseline in discovery sample	<0.001
		CpG13	PD < HC, sig. MAOA hypomethylation at baseline in discovery sample	<0.001

*iOFC* inferior orbitofrontal cortex, *BAT* Behavioral avoidance test.

Neuropeptide S is a neurotransmitter, it operates as a neuromodulator, especially in the onset of anxiety and arousal [33]. The T allele of NPSR1 SNP is associated with panic disorder and elevated heart rate [34]. Two studies explored the functional A/T single nucleotide polymorphism (SNP) r324981 in NPSR1. In the first one, the modulation of fear-related amygdala responsiveness in healthy controls (HC) associated with the NPSR gene, rs324981 A/T was investigated [35]. T-allele carriers had an increase in amygdala responsiveness to fear-relevant faces and harm avoidance. In the second one, the association of the NPSR1 T-risk allele with malfunctioning in the front-limbic network during anticipation and perception of agoraphobia-specific stimuli in PD/AG patients and HCs was explored [36]. PD/AG patients who were T-carriers showed higher amygdala and inferior orbitofrontal cortex (iOFC) activation in response to agoraphobic stimuli compared to neutral images compared to the HC group.

Serotonin-1A receptor (5-HTR1A) plays an important role in the regulation of the brain serotonin system [37]. Disturbances in 5-HTR1A functions may participate in pathogenesis of PD [38]. Recent studies of animal models have demonstrated that disturbances in 5-HTR1A functions contribute to anxiety-like behaviors [39]. Three studies investigated the HTR1A gene, specifically the rs6295 polymorphism. PD patients without agoraphobia exhibited higher Panic Disorder Severity Scale (PDSS) scores in the GG genotype than those with the CC genotype [40]. Patients with the G-allele exhibited significantly higher PDSS scores than those with the C-allele. In addition, there was a positive correlation between higher Albany Panic and Phobia Questionnaire (APPQ) interoceptive fear subscale scores and the GG/CG genotype [41]. The CC genotype positively correlates with APPQ, APPQ agoraphobia, and APPQ social phobia. One hypothesis is GG genotype carriers in PD, particularly in PD/AG, show a significant positive correlation with acute flight behavior before therapy compared to CC genotype carriers [42]. Significant interaction effects were noted between genotype (GG and CC carriers) and behavior (escapers vs. non-escapers) on reported anxiety during anticipation and exposure periods.

Several studies point the involvement of the norepinephrine system in the pathogenesis of PD [43]. The NET gene (SLC6A2 for solute carrier 6 family member 2) has been suggested as a candidate gene for other mental disorders like attention deficit hyperactivity disorder and depression [44]. Associations between the SLC6A2 gene and (PD) were demonstrated, with polymorphisms such as rs2242446, rs11076111, rs747107, rs1532701, rs933555, rs16955584, and rs36021 [45]. Notably, two marker haplotypes, T-T and C-T, were identified, with T-T serving as a protective haplotype and C-T as a risk haplotype. Single nucleotide polymorphisms (SNPs) of microRNA genes that regulate regulating SLC6A2 expression were investigated. The rs2910931 polymorphism in the MIR579 gene was associated with PD/AG and higher trait anxiety in healthy individuals [46].

One potential genetic risk factor for anxiety disorders is the neuronal nitric oxide synthase gene (NOS1). NOS1 encodes the neuronal isoform of nitric oxide synthase (NOS-I) which catalyzes the production of the neurotransmitter nitric oxide (NO) and is expressed throughout the brain [47]. Zebrafish and mice showed increased anxiety-like behavior under decreased NO signaling [48]. The functional promoter polymorphism of Nitric Oxide Synthase 1 (NOS1) gene was evaluated, specifically the NOS1 ex1f-VNTR short (S) allele. This study revealed significant interactions with the bilateral hippocampus and left amygdala in PD/AG patients during fear conditioning and extinction tasks [49]. An interaction effect between diagnosis and genotype unveiled distinct activation patterns in PD/AG patients compared to healthy controls.

Some studies suggest that polymorphisms in angiotensin-related genes affect anxiety disorders such as PD [50]. ACE has been shown to degrade a neurotransmitter called substance P (SP) [51]. Increase in SP concentration produces anxiogenic-like responses in rats [52]. In addition, the human angiotensin II type I receptor (ATr1) gene encodes ATr1, which is activated by angiotensin II in the brain [53]. The single nucleotide polymorphism (SNP) A1166C in the ATr1 gene was hypothesized to be involved in PD pathogenesis [50]. A study analyzed the I/D polymorphism in the ACE gene and the A1166C polymorphism in the ATR1 gene [54]. While no significant differences in genotype frequencies were discerned between the PD and healthy control groups, the male subgroup of PD patients exhibited a higher frequency of the I-allele ACE I/D polymorphism compared to controls.

The monoamine oxidase A (MAOA) is a key enzyme in the degradation of biogenic amines such as serotonin and dopamine. MAO inhibitors such as phenelzine or moclobemide are effective in the treatment of PD [55]. On a genetic level, the more active longer alleles of a functionally relevant 30 bp variable number tandem repeat (VNTR) in the MAOA gene (Xp11.4–p11.3) have repeatedly been found to be associated with PD, specifically in the female patients [56]. Epigenetic processes such as methylation of the cytosine pyrimidine ring in cytosine/guanine (CpG) dinucleotides critically influence gene expression, with methylation mainly “silencing” DNA transcription [57]. TMAOA gene and CpG methylation were also examined in all-female samples of PD patients across a discovery and replication sample, incorporating cognitive-behavioral therapy (CBT) treatment for the PD group [58]. In the discovery sample, hypomethylation at specific CpG sites was observed in PD patients compared to HC. Responders to treatment exhibited higher methylation averages at certain CpG sites compared to non-responders.

Putting together, these studies investigate genotypes, allelic variations, and gene methylation. Key genes studied include NPSR1, HTR1A, and SLC6A2. Studies on NPSR1 explored the rs324981 SNP, finding T-allele carriers had increased amygdala responsiveness and activation in response to agoraphobic stimuli. HTR1A studies examined the rs6295 polymorphism, noting higher PDSS scores in GG genotype carriers and correlations with anxiety. SLC6A2 gene associations with PD were demonstrated, identifying protective and risk haplotypes. Additionally, studies on NOS1, ACE, ATR1, and TMAOA genes, along with CpG methylation, provide further insights into genetic and epigenetic factors influencing PD susceptibility and treatment response.

[Note: The individual details of each study have been summarized for brevity.]

## Discussion

This systematic review has provided comprehensive insights into the neurochemical and genetic/epigenetic factors associated with the etiology and pathophysiology of Panic Disorder (PD). The reviewed studies have contributed valuable information across various domains, allowing for a qualitative synthesis of the most noteworthy findings.

A key neurochemical factor identified in the review is the role of Corticotropin-Releasing Hormone (CRH) in modulating the amygdala and the stress response. CRH, released in response to stress, influences the hypothalamic-pituitary-adrenal (HPA) axis, leading to the release of cortisol. Dysregulation of the HPA axis is implicated in anxiety and depressive disorders. CRH receptors are crucial for stress responses, and CRH1 receptor blockade or gene deletion has been associated with decreased defensive behaviors and attenuated stress responses. Additionally, single nucleotide polymorphisms (SNPs) in the CRH gene are linked to behavioral inhibition, a childhood risk factor for PD and social phobia. The interplay of CRH with various neurotransmitter systems, including glutamate, dopamine, serotonin, and norepinephrine, further underscores its significance in understanding PD pathophysiology.

Furthermore, neurotransmitters such as GABA and serotonin, with lower receptor binding in the amygdala, have been implicated in PD. Orexin (ORX) neurons in the dorsomedial/perifornical area have been identified as crucial for triggering panic reactions. Human subjects with panic anxiety exhibit elevated levels of ORX in cerebrospinal fluid, and ORX1 receptor antagonists have demonstrated efficacy in reducing panic responses.

The review also highlights the involvement of inflammatory markers, such as Interleukin 6 (IL-6) and leptin, in PD and Major Depressive Disorder (MDD) patients. Stress-induced inflammatory changes in the immune system, activating proinflammatory cytokines, are considered relevant in PD. Additionally, altered T cell populations have been associated with higher levels of anxiety in PD patients.

On the genetic and epigenetic front, the neuropeptide S receptor gene (NPSR1), 5-Hydroxytryptamine Receptor 1A (HTR1A) gene, and the norepinephrine transporter gene SLC6A2 emerge as key players in PD. Polymorphisms in these genes have been linked to various aspects of PD, including fear responsiveness, amygdala activation, and the stress network. The review emphasizes the significance of understanding genetic variations and epigenetic modifications in unraveling the complex etiology of PD.

In conclusion, this systematic review has synthesized a wealth of information regarding the intricate interplay of neurochemical and genetic/epigenetic factors in PD. The findings underscore the multifaceted nature of PD pathophysiology, providing a foundation for further research and targeted interventions. Integrating knowledge from diverse studies, the review sheds light on potential avenues for future investigations and therapeutic approaches in the realm of panic disorder.

### Strengths and limitations of this study

This study provides a comprehensive exploration of various facets of Panic Disorder (PD) pathophysiology, addressing a notable gap in the existing literature by systematically integrating neurochemical and genetic/epigenetic data. The methodology employed in this systematic review has enabled a thorough examination of the available evidence, offering valuable insights into the complex interplay of factors contributing to PD.

While the data presented in this study cover a wide range of topics, it is acknowledged that the heterogeneity of the available studies precluded the possibility of conducting a meta-analysis. A meta-analysis would have allowed for a quantitative synthesis of the findings, enhancing the precision and generalizability of the results. The acknowledgment of this limitation reflects the commitment to transparency and scientific rigor in the review process.

Moreover, the study rightly emphasizes the need for more research endeavors that integrate neuroanatomical, neurochemical and, genetic/epigenetic outcomes. This call for further studies is crucial for developing a more comprehensive and cohesive understanding of the alterations occurring in these diverse areas in the context of PD. The integration of findings across these domains can potentially unveil intricate relationships and mechanisms, providing a more holistic perspective on the pathophysiology of PD.

In conclusion, this systematic review not only contributes significantly to the current understanding of PD but also highlights the directions for future research. The call for more integrated studies across multiple domains indicates a commitment to advancing the field and addressing the complexities of PD pathophysiology. This study serves as a foundation for future investigations that aim to unravel the nuanced interconnections between neurochemical and genetic/epigenetic factors in the context of PD.

### Implications for research

To comprehensively understand the associations between neuroanatomical, neurochemical, genetic, and epigenetic data and their relevance to the neurobiology of Panic Disorder (PD), several critical steps and avenues for future research are essential.

**Central Fear Circuitry and Limbic Network:** Much of the existing research has focused on the dysregulation of central fear circuitry, particularly within the limbic network. This involves investigating the connections between key regions such as the amygdala, anterior cingulate cortex, and periaqueductal gray (PAG) during panic symptoms. Further exploration of these connections and their modulation during panic episodes is crucial for unraveling the neurobiological basis of PD.
**Blood-Brain Barrier and Connectivity:** Exploring the potential role of areas lacking a blood-brain barrier is a significant avenue for investigation. Understanding their connectivity to downstream sites responsible for behavioral and physiological responses during panic symptoms is vital. This exploration may provide insights into the involvement of non-traditional brain regions in the manifestation of PD.
**Animal Studies and Translational Models:** Animal studies have been instrumental in informing our understanding of PD’s etiology, mechanisms, and the role of genetic/epigenetic factors in fear circuitry. However, refining translational models is imperative to understand PD in humans. Developing more sophisticated models will contribute to a more accurate representation of the molecular and neural systems involved in PD.
**Technological Advances in Neuroimaging:** Future research directions should leverage technological advances in neuroimaging techniques. Incorporating state-of-the-art imaging methods will enhance our ability to investigate neuroanatomical, neurochemical, genetic, and epigenetic factors simultaneously. This multi-modal approach can provide a more comprehensive understanding of the intricate neural networks associated with PD.
**Human Research Integration:** Integrating neuroanatomical, neurochemical, genetic, and epigenetic factors in human research is essential. Collaborative studies that combine these different dimensions will contribute to a more holistic understanding of PD’s pathophysiology in the context of the human brain.
**New Treatments and Patient Outcomes:** The ultimate goal is to translate research findings into new treatments for PD patients. A deeper understanding of the neurobiological underpinnings can inform targeted interventions to alleviate the debilitating effects and burden of PD on individuals.
**Fundamental Mechanistic Research:** Future studies should delve into fundamental mechanistic research to understand the integrative role of neuroanatomical, neurochemical and, genetic data in PD. Investigating the crosstalk among these factors and their neuromodulatory functions will provide crucial insights into the pathophysiological mechanisms of PD.

In summary, advancing our understanding of PD requires a multidimensional approach that integrates findings from various disciplines. By combining neuroanatomical, neurochemical, genetic, and epigenetic data, researchers can uncover the intricate web of factors contributing to PD. This comprehensive understanding is pivotal for developing effective treatments and improving outcomes for individuals affected by PD.

## Conclusion

This review has systematically presented a collection of studies investigating alterations in neurochemical, genetic/epigenetic, and animal data in the context of Panic Disorder (PD). The cumulative findings from these studies reveal discernible patterns of dysregulated expression in various biological systems, encompassing neurotransmission, the Hypothalamic-Pituitary-Adrenal (HPA) axis, neuroplasticity, as well as genetic and epigenetic factors, which in turn result in neuroanatomical modifications.

The intricate nature of neuropsychiatric illnesses, particularly Panic Disorder, is closely linked to the abnormal functioning of neural circuits. These circuits involve multiple brain regions, each contributing to distinct features of the disorder. As demonstrated in the literature, different sets of neural circuits are responsible for various types of defensive responses. Therefore, a comprehensive understanding of the brain regions involved, and their functional connectivity is crucial for unraveling the neurobiological foundation of PD.

The recognition that complex emotional and cognitive processing is intricately tied to neural circuit functioning emphasizes the need to explore the functional connectivity between different brain regions. This knowledge could significantly enhance our understanding of PD’s neurobiological underpinnings. Moreover, it lays the groundwork for the development of effective interventions that span diverse modalities, including pharmacological approaches, psychotherapeutic interventions, environmental considerations, and lifestyle modifications.

In essence, the synthesis of findings presented in this review not only contributes to our current understanding of PD but also highlights the potential avenues for future research and intervention strategies. By delving into the intricate interplay of neurochemical and genetic/epigenetic by exploring the functional connectivity of neural circuits, this review provides a foundation for advancing our comprehension of the multifaceted nature of Panic Disorder and, consequently, for devising more targeted and comprehensive therapeutic approaches.
